# PI3Kγ Deficient NOD-Mice Are Protected from Diabetes by Restoring the Balance of Regulatory to Effector-T-Cells

**DOI:** 10.1371/journal.pone.0169695

**Published:** 2017-01-12

**Authors:** Jamil Azzi, Lindsay Thueson, Robert Moore, Rozita Abdoli, Helena Reijonen, Reza Abdi

**Affiliations:** 1 Transplantation Research Center, Renal Division, Brigham and Women's Hospital, Harvard Medical School, Boston, Massachusetts, United States of America; 2 Benaroya Research Institute, Seattle, Washington, United States of America; Children's Hospital Boston, UNITED STATES

## Abstract

With a steady increase in its incidence and lack of curative treatment, type 1 diabetes (T1D) has emerged as a major health problem worldwide. To design novel effective therapies, there is a pressing need to identify regulatory targets controlling the balance of autoreactive to regulatory-T-cells (Tregs). We previously showed that the inhibition of the γ-subunit of the Phosphoinositide-3-kinase (PI3K), significantly suppress autoimmune-diabetes. To further delineate the mechanisms and the selectivity of specific immune modulation by PI3Kγ-inhibition, we developed a new NOD mouse model of T1D lacking the γ-subunit of PI3K. Strikingly, the loss of PI3Kγ protected 92% of the NOD-mice from developing spontaneous diabetes. The NOD.PI3Kγ^-/-^ mice are protected from insulitis secondary to a defect in CD4 and CD8 autoreactive-T-cells activation and survival. In addition, PI3Kγ-deficiency promoted Treg generation *in-vitro* and *in-vivo*. Furthermore, PI3Kγ-inhibitor (AS605240) inhibited proliferation and cytokine production of a human CD4^+^ T-cell clone specific for GAD555-567 peptide that was isolated from a patient with T1D. These studies demonstrate the key role of the PI3Kγ pathway in regulating autoimmune-diabetes and provide rationales for future devise of anti- PI3Kγ therapy in T1D.

## Introduction

Type-1-diabetes (T1D) remains a major health problem with steadily increasing incidence, yet no cure[1]. An immune balance is established after birth in which islet autoreactive-T-cells are kept in check by immune regulators such as Regulatory T-cells (Tregs), preventing these cells from becoming active[2]. While current evidence shows that T1D develops due, at least in part, to a defect in Tregs function, identifying new signaling-pathways that preferentially control the survival of these autoreactive-T-cells while sparing Tregs is crucial to the development of therapeutic-strategies for T1D[3,4].

We have recently shown that the Phosphoinositide-3-kinases (PI3Ks), a family of both lipid and protein-kinases involved in multiple intracellular functions, is highly activated in splenocytes of NOD-mice compared to diabetes-resistant-mice[5,6]. While Phosphorylated-Akt (pAkt), a secondary messenger of PI3K, was shown to promote cell survival[6], we found that pAkt is highly expressed in effector-T-cells compared to Tregs[5]. This differential expression of pAkt suggested a differential role of PI3K in effector-T-cells versus Tregs survival and raised our interest in studying this pathway in T1D.

Although PI3K-molecules have long been regarded as promising drug targets for the treatment of inflammatory and autoimmune-disorders, the wide tissue distribution of PI3K-expression hindered any significant advancement of drug therapy. Discovery of the PI3K-subunits, in particular the leukocyte-restricted γ-subunit are promising advances in the efforts to validate PI3K as a safe therapeutic target for autoimmune-diseases[7–9].

The PI3Ks are classified as class-IA and IB, according to their mode of activation: class-IA PI3Ks are activated downstream of tyrosine kinase receptors, whereas class-IB PI3K has only one subunit (PI3Kγ) and is activated by G-protein-coupled-receptors.

Genetically targeting the p110 catalytic subunit of PI3Kγ revealed a critical role for this pathway in the activation of mature T cells and neutrophils migration and respiratory burst in response to chemotactic agents. Homozygous PI3Kγ^-/-^ mice were born at the expected Mendelian ratio, appeared healthy, and were fertile[8,10].

Here, we sought to test whether PI3Kγ is required for spontaneous autoimmune-diabetes by backcrossing PI3Kγ^-/-^ C57BL/6 mice with the NOD-mice. Our results show that the loss of PI3Kγ protects 92% of the NOD-mice from developing spontaneous diabetes. The NOD. PI3Kγ^-/-^ mice are protected from insulitis and show a defect in effector-T-cell function and survival. In addition, PI3Kγ-deficiency promotes Treg generation *in-vitro* and *in-vivo*. Furthermore, we tested the effect of PI3Kγ-inhibitor (AS605240) on a human CD4^+^ T-cell clone specific for GAD555-567 peptide that was isolated from a patient with T1D. Proliferation and cytokine production of the T-cell clone was significantly reduced when cultured in the presence of AS605240, even at low concentrations of the drug.

Our data highlight the critical role of PI3Kγ in T1D and support the concept of translating PI3Kγ-inhibition therapy to patients with T1D.

## Materials and Methods

### Mice

Homozygous NOD.PI3Kγ^-/-^, heterozygous NOD.PI3Kγ^-/+^ and WT NOD.PI3Kγ^+/+^ littermates were bred and housed in a specific pathogen-free barrier facility at Harvard Medical School.

PI3Kγ^-/-^ mice on the C57BL/6 background are a generous gift from Dr. Lu[11]. NOD/ShiLtJ mice were purchased from Jackson Laboratory. The Harvard Medical School Animal Management Committee approved all animal experiments. Diabetes monitoring started at age of 8 weeks for all mice with 2 measurements per week. Mice with blood sugar above 250 mg/dl for 2 consecutive days were considered diabetic. Number of mice monitored (WT NOD.PI3Kγ^+/+^ littermates (16 mice); Homozygous NOD.PI3Kγ^-/-^ (13 mice); heterozygous NOD.PI3Kγ^-/+^ (5 mice)).

### Flow cytometric analysis

Anti-mouse Abs against CD62L, CD44, CD4, CD8, Annexin were purchased from BD Biosciences (San Jose, CA), and Tregs were assessed using CD4, CD25 and FoxP3 (eBioscience). Cells recovered from lymphoid tissues were analyzed using a FACS Canto-II flow cytometer (BD Biosciences) and analyzed using FlowJo software version 9.3.2 (Tree Star, Ashland, OR).

### Autoreactive-T-cell proliferation assay

5x10^5^ NOD splenocytes and 50 μg/ml BDC2.5-peptide (Ac-RTRPLWVRME amide, QCB, Hopkinton MA), 50 μg/ml IGRP^(206–214)^ peptide (VYLKTNVFL) were incubated *in-vitro* in a 96-well-round-bottom-plate for 48 hours. Cultures were pulsed with 1 μCi of tritiated-thymidine-(3H) to determine cell-proliferation.

### Luminex-assay

Cell-free supernatants of individual wells of the autoreactive T cell assay were removed after 48 h of incubation and analyzed by a multiplexed cytokine bead–based immunoassay using a preconFigd 21-plex mouse cytokine detection kit (Millipore, St. Charles, MO). All samples were tested in triplicate wells.

### Pancreas pathology

Each formalin-fixed and paraffin-embedded specimen was cut into sections 5 μm thick. Staining was performed with hematoxylin-eosin (H&E) for routine histological observations. A score of 0 to 4 was assigned based on islet-infiltration by a blinded pathologist[5]. At least 30 islets per group were analyzed from at least 4 different mice.

### Statistical analyses

Data are expressed as mean ± standard-error. Kaplan-Meier analysis was used for survival analysis. The Mann-Whitney test was used for comparison of means between experimental groups. p less than 0.05 was considered significant.

### Human autoreactive-T-cell clone proliferation assay: PI3Kγ-inhibitor dose-response titrations

HLA-DR0401^+^ PBMCs were thawed, rested and pulsed with 20μg/mL peptide GAD555-567(557I) at 37°C. The following day, PBMC were irradiated, and cultured with a T-cell-clone CGad73 at final concentration of 10ug/mL of the peptide GAD555-567(557I) and at increasing concentrations (2.5-50uM) of PI3Kγ-Inhibitor, AS605240. The T-cell-clone was isolated from DR401-positive T1D patient who was recruited at the Diabetes-Clinical-Research Unit at Benaroya-Research-Institute. The subject was HLA-DR401^+^ (DRB1*0401, DQB1*0302/DRB1*0101, DQB1*0501) and positive for GAD65-autoantibodies at the time the blood sample was drawn. The PBMC were stimulated with GAD65 555–567 (557I, agonist peptide) peptide as previously described [12]. Cells were suspended in 200μL media at a 1:3 ratio (0.5x10^5^clones:1.5x10^5^PBMC/well) and incubated at 37°C in a 96-well-flat-bottom-plate in triplicate-wells. At day 3, cells were pulsed with 1μCi of tritiated-thymidine (^3^H) for 8 hours, and harvested onto a glass fiber filter. Proliferation was measured through liquid scintillation-counting, 1 minute/well.

## Results

### NOD.PI3Kγ^-/-^ mice are protected from diabetes

**Fig 1:**
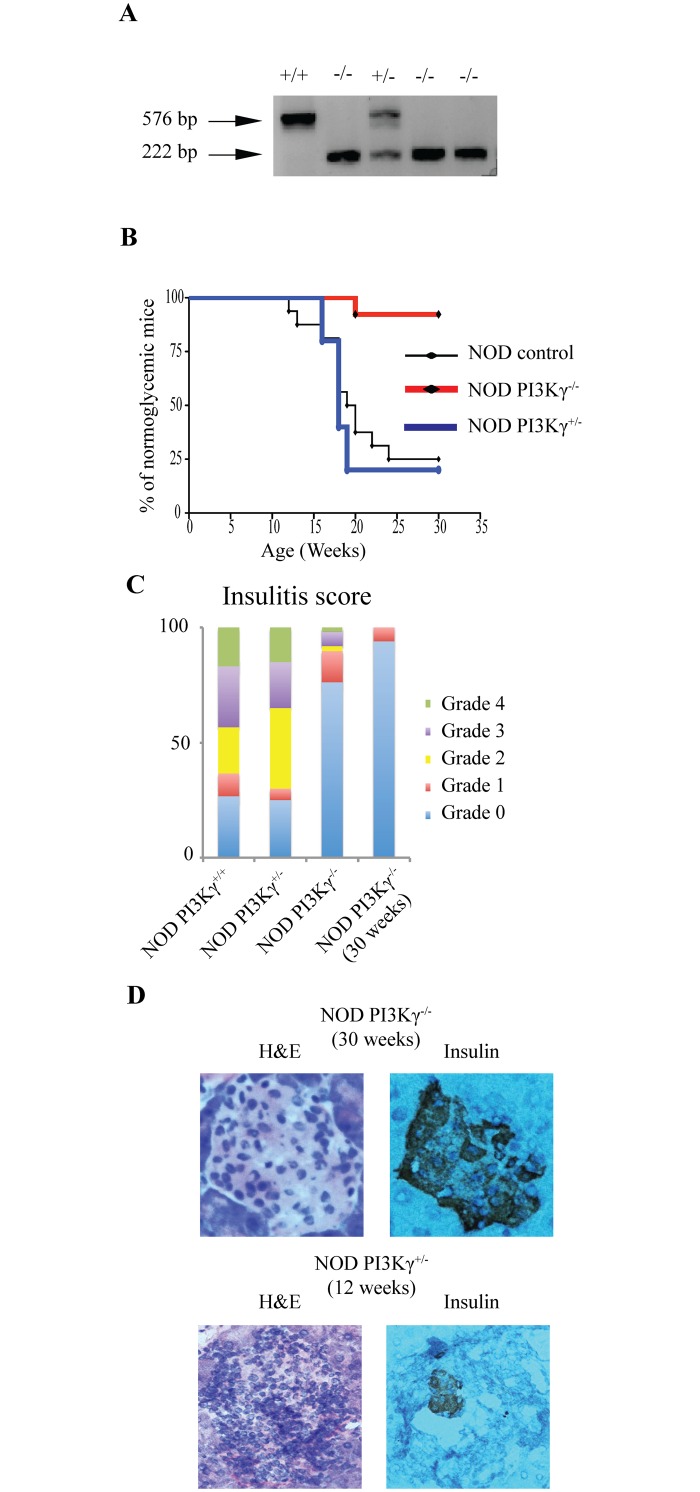
PI3Kγ-deficiency protects NOD-mice from diabetes. (A) PCR bands of WT NOD.PI3Kγ^+/+^(576 bp), homozygous NOD.PI3Kγ^-/-^ (222 bp) and heterozygous NOD.PI3Kγ^-/+^ (222 bp and 576 bp). (B) Female NOD.PI3Kγ^-/-^, their heterozygous-NOD.PI3Kγ^+/-^ and WT-NOD.PI3Kγ^+/+^ littermates were followed for diabetes for 30-weeks. Graph represents the percentage of mice developing diabetes overtime. (C) Insulitis score at 12-weeks of age measured on 30 different islets from 4 mice per group is presented in the panel 1C. Mice from the above cross were sacrificed for histopathological analysis of the pancreas at age of 12-weeks. grade 0, normal islets; grade 1, mild mononuclear infiltration (<25%) at the periphery; grade 2, 25–50% of the islets infiltrated; grade 3, >50% of the islets infiltrated; grade 4, islets completely infiltrated with no residual parenchyma remaining. (D) Representative example of pancreatic histology (H&E and insulin staining) from NOD.PI3Kγ^+/-^ at 12-weeks and NOD.PI3Kγ^-/-^ at 30-weeks of age.

We generated NOD.PI3Kγ^-/-^ mice by backcrossing PI3Kγ^-/-^ mice on the C57BL/6-background (gift from Dr. Lu)[11], with NOD/ShiLtJ mice (Jackson Laboratory), using the Marker-Assisted-Accelerated-Backcrossing-program at Charles-River Laboratory (S1 File). A scan of a panel of 384 single-nucleotide-polymorphisms (SNP) throughout of the genome of NOD.PI3Kγ^-/-^ revealed that all chromosomes were NOD-derived except for part of chromosome-12, which contains the null-allele of PI3Kγ. NOD.PI3Kγ^-/-^ mice have an allele-deficiency within the PI3Kγ-gene located on chromosome 12 at 12;12B. Our search of 26 known insulin-dependent-diabetes (IDD) genes shows that PI3Kγ is not near any known IDD genes(S1 Table). Fig 1 shows the PCR analysis of tissues removed from WT-NOD.PI3Kγ^+/+^, homozygous-NOD.PI3Kγ^-/-^ and heterozygous-NOD.PI3Kγ^+/-^. To determine diabetes incidence, female offspring of NOD.PI3Kγ^+/-^, NOD.PI3Kγ^-/-^ and NOD.PI3Kγ^+/+^ mice were followed by blood glucose measurement for 30-weeks. NOD.PI3Kγ^-/-^ were 92% protected from diabetes, significantly different than NOD.PI3Kγ^+/-^ (p< 0.01). Similarly NOD.PI3Kγ^-/-^ were significantly more protected from diabetes than NOD.PI3Kγ^+/+^ (p< 0.01) that became diabetic with normal kinetics for our colony (Fig 1B). Histological analysis of pancreata showed that NOD.PI3Kγ^-/-^ females at 12-weeks of age are protected from insulitis compared to heterozygous-NOD.PI3Kγ^+/-^ and WT-NOD.PI3Kγ^+/+^. (Fig 1C). Histological analysis of pancreata from 30-weeks old NOD.PI3Kγ^-/-^ female, showed normal islets with no significant infiltrate (Fig 1D).

### PI3Kγ-deficiency reduces effector-T-cells and increases Tregs in NOD-mice

**Fig 2:**
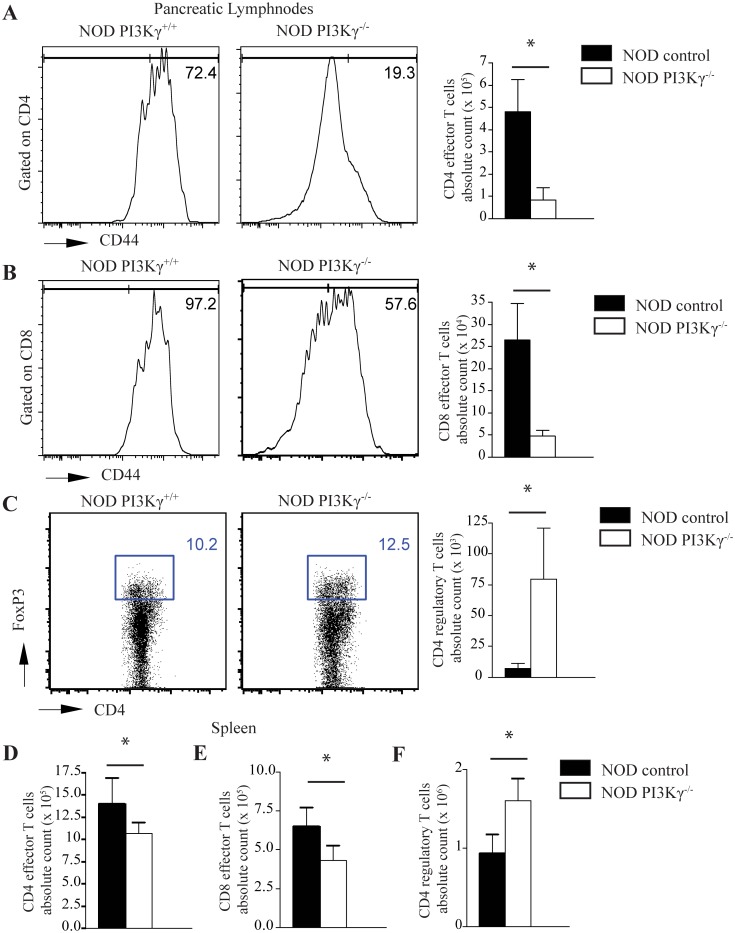
PI3Kγ-deficiency restores the balance of effector versus regulatory T-cells toward regulatory. (A) Representative example of FACS staining from pancreatic lymphnodes of NOD.PI3Kγ^+/+^ and NOD.PI3Kγ^-/-^ at 12-weeks of age shows lower percentage of CD4 effector-T-cells (CD4+CD44high) in mice lacking PI3Kγ compared to WT-NOD.PI3Kγ^+/+^ mice. Bar graph shows the absolute count of CD4 effector-T-cells. (*p<0.05, n = 4 mice in each group). Results are presented as the mean ± s.e.m. (B) Representative example of FACS staining from pancreatic lymphnodes of NOD.PI3Kγ^+/+^ and NOD.PI3Kγ^-/-^ at 12-weeks of age shows lower percentage of CD8 effector-T-cells (CD8+CD44high) in mice lacking PI3Kγ compared to WT-NOD.PI3Kγ^+/+^. Bar graph shows the absolute count of CD8 effector-T-cells. (*p<0.05, n = 4 mice in each group). Results are presented as the mean ± s.e.m. (C) Representative example of FACS staining from pancreatic lymphnodes of NOD.PI3Kγ^+/+^ and NOD.PI3Kγ^-/-^ at 12-weeks of age shows higher percentage of CD4 regulatory T-cells (CD4+FoxP3+) in mice lacking PI3Kγ compared to WT- WT-NOD.PI3Kγ^+/+^. Bar graph shows the absolute count of CD4 regulatory T-cells. (D) Bar graph shows the absolute count of CD4 effector-T-cells (CD4+CD44high) in splenocytes of mice lacking PI3Kγ compared to WT-NOD.PI3Kγ^+/+^. (E) Bar graph shows the absolute count of CD8 effector-T-cells (CD8+CD44high) in splenocytes of mice lacking PI3Kγ compared to WT- WT-NOD.PI3Kγ^+/+^. (F) Bar graph shows the absolute count of CD4 regulatory T-cells(CD4+FoxP3+) in splenocytes of mice lacking PI3Kγ compared to WT-NOD.PI3Kγ^+/+^ (*p<0.05, n = 4 mice in each group). Results are presented as the mean ± s.e.m.

Tregs play a critical role in controlling the balance between immune effector function and regulation in diabetes in NOD-mice [13]. Flow-cytometric analysis of pancreatic-lymphnodes of NOD.PI3Kγ^-/-^ and WT-NOD.PI3Kγ^+/+^ showed a decrease in the percentage and the absolute-number of effector CD4^+^ and CD8^+^ T-cells (CD4^+^CD44^high^ and CD8^+^CD44^high^ respectively) at 12-weeks of age in NOD.PI3Kγ^-/-^ compared to WT-NOD.PI3Kγ^+/+^ (Fig 2A and 2B). Furthermore, there was a significant increase in the percentage and absolute count of Tregs (CD4^+^FoxP3^+^) in the pancreatic-lymphnodes of NOD.PI3Kγ^-/-^ compared to WT-NOD.PI3Kγ^+/+^ (Fig 2C). Similar results were observed by flow-cytometry analysis of the spleens of NOD.PI3Kγ^-/-^ and WT-NOD.PI3Kγ^+/+^ (Fig 2D, 2E and 2F).

### PI3Kγ-deficiency suppresses autoreactive-T-cells proliferation and survival

**Fig 3:**
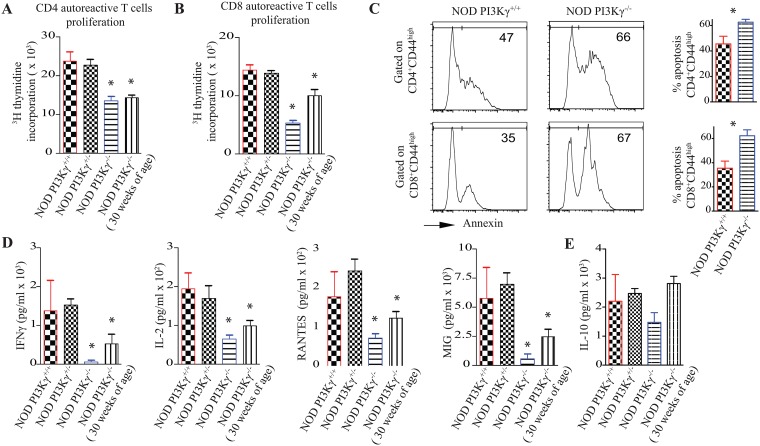
PI3Kγ-deficiency impairs autoreactive-T-cells activation and survival upon stimulation with pancreatic-peptides. (A) Graph represents autoreactive CD4 T-cell proliferation for 48 hours as measured by thymidine incorporation. Splenocytes recovered from NOD.PI3Kγ^+/+^, NOD.PI3Kγ^+/-^, NOD.PI3Kγ^-/-^ at 12-weeks of age and NOD.PI3Kγ^-/-^ at 30-weeks were stimulated *ex-vivo* with BDC2.5 pancreatic peptide. (B) Graph represents autoreactive CD8 T-cell proliferation as measured by thymidine incorporation. Splenocytes recovered from NOD.PI3Kγ^+/+^, NOD.PI3Kγ^+/-^, NOD.PI3Kγ^-/-^ at 12-weeks of age and NOD.PI3Kγ^-/-^ at 30-weeks were stimulated ex-vivo with IGRP pancreatic peptide. (C) Representative examples of apoptosis analysis by flow-cytometry on CD4 and CD8 effector-T-cells 2 days after stimulation with pancreatic-peptides. CD4 and CD8 effector-T-cells (CD44high) of NOD.PI3Kγ^-/-^ went more into apoptosis as measured by annexin expression compared to WT-NOD.PI3Kγ^+/+^. (D and E) Graphs represent cytokines analysis as measured by Luminex-assay on supernatant from an *ex-vivo* BDC2.5 pancreatic peptide challenge of splenocytes recovered from the different mice. (*p<0.05, n = 4 mice/group). As shown in panels A and B autoreactive-T-cells from the NOD.PI3Kγ^-/-^ proliferated less and produced less inflammatory cytokines compared to NOD.PI3Kγ^+/+^, NOD.PI3Kγ^+/-^. (*p<0.05, n = 4 mice/group).

To assess the functional role of PI3Kγ in the activation and survival of autoreactive-T-cells, splenocytes from 12 and 30-weeks old NOD.PI3Kγ^-/-^ mice, 12-weeks old heterozygous-NOD.PI3Kγ^+/-^ and WT-NOD.PI3Kγ^+/+^. were stimulated *ex-vivo* with pancreatic-peptides. We first analyzed the proliferation of autoreactive CD4^+^ T-cells *ex-vivo* by measuring thymidine-incorporation of splenocytes after BDC2.5-pancreatic-peptide challenge [5]. Splenocytes from 12 and 30-weeks old NOD.PI3Kγ^-/-^ mice proliferated significantly less than 12-weeks old heterozygous-NOD.PI3Kγ^+/-^ and WT-NOD.PI3Kγ^+/+^ (Fig 3A). Similarly, autoreactive CD8^+^ T-cells from 12 and 30-weeks old NOD.PI3Kγ^-/-^ mice proliferated significantly less compared to WT-NOD.PI3Kγ^+/+^ upon stimulation of splenocytes with IGRP-pancreatic-peptide challenge (Fig 3B).

PI3K-pathway is one of the major cell-survival-pathways[14]. Therefore, to study the role of PI3Kγ pathway in T-cells survival, splenocytes from NOD.PI3Kγ^-/-^ and WT-NOD.PI3Kγ^+/+^ were stimulated *ex-vivo* with either BDC25 or IGRP pancreatic-peptides for 48 hours as described above. By flow-cytometry, we gated on activated NOD.PI3Kγ^-/-^ CD4^+^ and CD8^+^ T-cells as measured by expression of CD44^high^. Our data show that NOD.PI3Kγ^-/-^ CD4^+^CD44^high^ and CD8^+^CD44^high^ T-cells went more into apoptosis, as measured by Annexin expression (apoptosis marker) compared to WT-NOD.PI3Kγ^+/+^ CD4^+^CD44^high^ and CD8^+^CD44^high^ T-cells (For CD4: 62.60 ± 2.295 vs. 45.53 ± 6.044 respectivly, p< 0.05; for CD8: 62.27 ± 4.934 vs. 35.47 ± 5.805 respectively, p = 0.01)(Fig 3C).

Furthermore, we assessed the activity of autoreactive-CD4^+^ T-cells by measuring the cytokine-patterns after a BDC2.5-pancreatic-peptide challenge of splenocytes. Supernatant from BDC2.5-peptides stimulated splenocytes was subjected to a luminex-assay. As shown in Fig 3D, splenocytes from 12 and 30-weeks old NOD.PI3Kγ^-/-^ mice produced less inflammatory-cytokines such as IFN-γ, IL-2, and less chemokines such as RANTES and MIG compared to 12-weeks old heterozygous-NOD.PI3Kγ^+/-^ and WT-NOD.PI3Kγ^+/+^. Interestingly, IL-10 production did not follow the same pattern as the other cytokines. IL-10 production by splenocytes from 12 and 30-weeks old NOD.PI3Kγ^-/-^ mice was not statistically significantly different from WT NOD splenocytes (Fig 3E).

### PI3Kγ-deficiency enhances Tregs generation *in-vitro*

We then assessed the effects of PI3Kγ-deficiency on Treg induction *in-vitro*. 2 x 10^5^ CD4^+^ CD25^-^ T-cells from NOD-mice were cultured for 72-hours in a CD3/CD28 stimulation-assay in the presence of 2 ng/ml TGF-β and 10 ng/ml of IL2. As shown in S1 Fig, flow-cytometric-analysis for CD4^+^CD25^+^FoxP3^+^ revealed significant induction of FoxP3^+^ cells from NOD.PI3Kγ^-/-^ compared to NOD.PI3Kγ^+/+^ CD4^+^ T-cells as shown in percentage (35.99 ± 2.700% vs. 25.60 ± 2.354%, respectively, p = 0.004).

### PI3Kγ-inhibitor (AS605240) suppresses human autoreactive-T-cell activation

**Fig 4:**
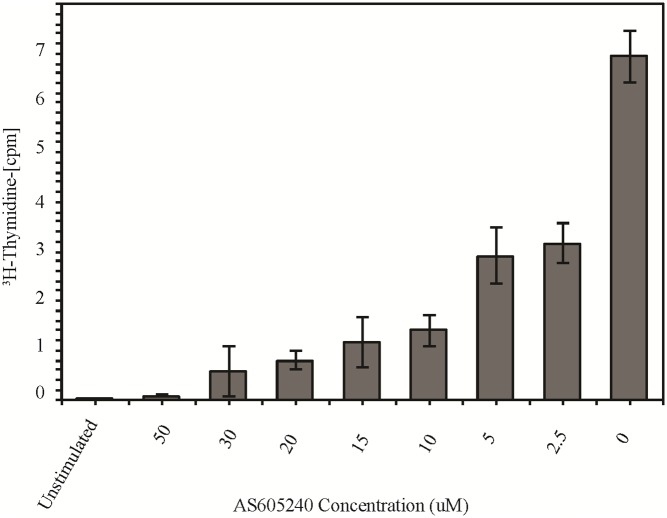
PI3Kγ-inhibitor (AS605240) suppresses human autoreactive-T-cell activation. T-cell clone C-Gad 73 was stimulated for 72 hours with irradiated HLADR401^+^ PBMC pulsed with GAD65 peptide in the presence of different concentrations of AS605240 and without the drug. Unstimulated control represents the latter condition without the peptide. Tritiated thymidine was added for the last 8 hours and proliferation of the T-cell clone was determined. The columns represent the means +/- SE of three wells per AS605240 concentration (μM). The y- axis rpresents the average counts per minute (cpm) of the DNA incorporation of tritiated (^3^H)-thymidine. CPM are shown as 1/10,000 of actual count (x10e4).

Finally, we determined whether inhibition of PI3Kγ by a specific PI3Kγ-inhibitor would affect T-cell activation and proliferation of human CD4^+^ T-cells. In this experiment we used a T-cell clone derived from a DR401^+^ T1D patient that was specific for an immunodominant-epitope from a human T1D autoantigen, GAD65. T-cells were stimulated in the presence of HLA-matched (DR401^+^) antigen-presenting-cells and a specific GAD65-peptide at different concentrations of the PI3Kγ-inhibitor, AS605240. As shown in Fig 4 proliferation of the T-cell-clone was significantly reduced when cultured in the presence of AS605240, even at low concentrations of the drug suggesting relevance of this pathway in T-cell activation and proliferation in human autoreactive-T-cells.

## Discussion

Pancreas and islet transplantation are currently available strategies to treat patients with T1D. However, each of these strategies carries its own risks and complications [15,16]. Hence, the need is to find a cure for the disease[17,18]. PI3K has risen to the forefront of drug-development in cancer and immunity, with the first clinical-trials of PI3K-inhibitors now in progress (clinicaltrials.gov). In this regard, PI3K-pathway is an attractive target for T1D-therapy. Among the various classes of PI3Ks, PI3Kγ expression is restricted to the hematopoietic-system and constitutes an attractive-target to treat inflammatory-diseases [9].

We have recently shown that inhibiting PI3Kγ using a small molecule (AS605240) prevented and reversed diabetes in NOD-mice[5]. The PI3Kγ-inhibitor potently abrogated autoreactive-T-cells while sparing Tregs. Furthermore, AS605240 increased the ratio of Tregs to effector populations in the treated mice, tipping the balance from autoreactive-T-cells to Tregs, thus conferring protection against autoimmune-diabetes[5].

The design of selective PI3Kγ-inhibitors was shown to be challenging. While the side chains of the amino acids, that distinguish the different PI3K-isoforms from each other, constitute an attractive-target for specific-inhibitors, they are difficult to reach with small molecules[7]. Hence, despite the advancements in developing PI3Kγ-inhibitors, the selectivity of the available compounds to the γ-subunit can’t be accurately determined. The data from our PI3Kγ-deficient NOD-mice reproduce the results from our previous work. Those data combined show that PI3Kγ-inhibition has the potential to cure T1D.

Furthermore, our data highlight the key role PI3Kγ-inhibition in restoring the balance of effector to regulatory T-cells toward regulatory, thus protecting from T1D. While we previously showed that pAkt, the secondary messenger of PI3K is highly expressed in effector-T-cells compared to Tregs, we suggested a differential role of PI3K in effector versus regulatory T-cells survival[5]. We show here an increased apoptosis of activated autoreactive CD4 and CD8 T-cells deficient in PI3Kγ compared to WT-NOD.PI3Kγ^+/+^. Furthermore, recent data showed that mutation of the PI3KR1 gene in a patient lead to reduced germinal center follicular helper T cells but spared follicular regulatory T cells in the tonsils[4].

Our data are in agreement with others who showed that the deletion of the PI3K-PKB pathway, downstream of PI3Kγ, decreases survival of pathogenic CD4^+^ memory cells in mouse models of SLE[19]. PI3Kγ has also been shown to play a critical role in the downstream signaling of TCR-mediated T-cell activation[20]. This is also supported by our data showing that the treatment of human autoreactive-T-cell clone with a low concentration of AS605240 (2.5uM) reduces the antigen induced activation by ~50%. We are in the process of examining the potentially distinct effect of this PI3Kγ-inhibitor on primary CD4^+^ effector-T-cells and Tregs in T1D patients as was described in NOD-mice.

In conclusion, these data support the critical role PI3Kγ in T1D and support the concept of translating PI3Kγ-inhibition therapy to patients with T1D.

## Supporting Information

S1 Fig PI3Kγ-deficiency enhances regulatory T-cells generation *in-vitro*. Representative Figs of flow-cytometry analysis for CD4^+^CD25^+^FoxP3^+^ in a Treg generation assay using NOD.PI3Kγ^-/-^ compared to NOD.PI3Kγ^+/+^ CD4^+^ CD25^-^ T-cells. 2 x 10^5^ CD4^+^ CD25^-^ T-cells were cultured for 72 hours in a CD3/CD28 stimulation assay in the presence of 2 ng/ml TGF-β and 10 ng/ml of IL2. As shown here, flow cytometric analysis for CD4^+^CD25^+^FoxP3^+^ revealed significant induction of FoxP3^+^ cells from NOD.PI3Kγ^-/-^ compared to NOD.PI3Kγ^+/+^ CD4^+^ T-cells. Graph represents the percentage of Tregs.
(PDF)

S1 Table List and location of known insulin-dependent diabetes (Idd) genes compared to the PI3Kγ gene. This table shows our search of the location of 26 known Idd genes in addition to the PI3Kγ gene (chromosome 12 at 12;12B). The PI3Kγ gene is not near any known Idd genes. This eliminates the possibility that the knockdown of the PI3Kγ gene might affect an adjacent IDD gene, known to control the development of autoimmune-diabetes in both humans and NOD-mice.
(PDF)

S1 File Marker-Assisted Accelerated Backcrossing supporting information. A. Description of the Marker-Assisted Accelerated Backcrossing technology used to generate the new NOD.PI3Kγ^-/-^ mouse. B. Data from the Marker-Assisted-Accelerated-Backcrossing-program at Charles-River Laboratory that shows the scan of a panel of 384 single-nucleotide-polymorphisms (SNP) throughout of the genome of NOD.PI3Kγ^-/-^ mouse.
(PDF)
