# Supplementary figures and images for: PI3Kγ Deficient NOD-Mice Are Protected from Diabetes by Restoring the Balance of Regulatory to Effector-T-Cells
Source: PLoS One. 2017 Jan 12;12(1):e0169695. doi: 10.1371/journal.pone.0169695 (PMC5231340; doi:10.1371/journal.pone.0169695)

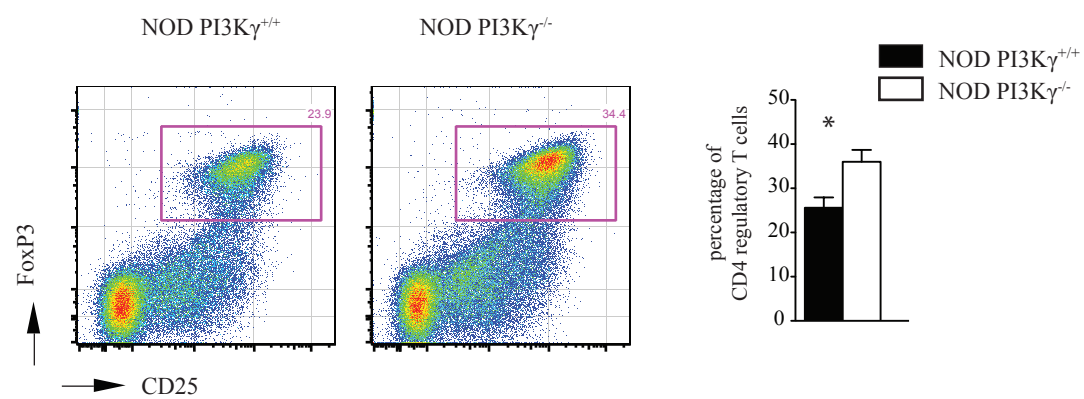

Azzi et al. Supplementary Figure 1

Supplement: S1 Fig — Representative Figs of flow-cytometry analysis for CD4+CD25+FoxP3+ in a Treg generation assay using NOD.PI3Kγ-/- compared to NOD.PI3Kγ+/+ CD4+ CD25- T-cells. 2 x 10^5^ CD4+ CD25- T-cells were cultured for 72 hours in a CD3/CD28 stimulation assay in the presence of 2 ng/ml TGF-β and 10 ng/ml of IL2. As shown here, flow cytometric analysis for CD4+CD25+FoxP3+ revealed significant induction of FoxP3+ cells from NOD.PI3Kγ-/- compared to NOD.PI3Kγ+/+ CD4+ T-cells. Graph represents the percentage of Tregs. (PDF) [file pone.0169695.s001.pdf]
