# Supplementary material for: PI3Kγ Deficient NOD-Mice Are Protected from Diabetes by Restoring the Balance of Regulatory to Effector-T-Cells
Source: PLoS One. 2017 Jan 12;12(1):e0169695. doi: 10.1371/journal.pone.0169695 (PMC5231340; doi:10.1371/journal.pone.0169695)
Supplement: S1 Table — This table shows our search of the location of 26 known Idd genes in addition to the PI3Kγ gene (chromosome 12 at 12;12B). The PI3Kγ gene is not near any known Idd genes. This eliminates the possibility that the knockdown of the PI3Kγ gene might affect an adjacent IDD gene, known to control the development of autoimmune-diabetes in both humans and NOD-mice. (PDF) [file pone.0169695.s002.pdf]

List of the known Idd genes

IDD gene	Location	Publication
Idd1	Chromosome 17	Lyons, Journal of Immunology, 1997
Idd2	Chromosome 9	http://www.ncbi.nlm.nih.gov/gene/?term=Idd2
Idd3	Chromosome 3	Lyons, Journal of Immunology, 1997
Idd4	Chromosome 11	http://www.ncbi.nlm.nih.gov/gene/110675
Idd5	Chromosome 1	http://www.ncbi.nlm.nih.gov/gene/110656
Idd6	Chromosome 6	http://www.ncbi.nlm.nih.gov/gene/107835
Idd7	Chromosome 7	http://www.ncbi.nlm.nih.gov/gene/110649
Idd8	Chromosome 14	http://www.ncbi.nlm.nih.gov/gene/15923
Idd9	Chromosome 4	http://www.ncbi.nlm.nih.gov/gene/110658
Idd10	Chromosome 3	Lyons, Journal of Immunology, 1997
Idd11	Chromosome 4	http://www.ncbi.nlm.nih.gov/gene/110713
Idd12	Chromosome 14	http://www.ncbi.nlm.nih.gov/gene/15909
Idd13	Chromosome 2	http://www.ncbi.nlm.nih.gov/gene/15910
Idd14	Chromosome 13	http://www.ncbi.nlm.nih.gov/gene/15911
Idd15	Chromosome 5	http://www.ncbi.nlm.nih.gov/gene/15912
Idd16	Chromosome 17	Lyons, Journal of Immunology, 1997
Idd17	Chromosome 3	http://www.ncbi.nlm.nih.gov/gene/15914
Idd18	Chromosome 3	http://www.ncbi.nlm.nih.gov/gene/110736
Idd19	Chromosome 6	http://www.ncbi.nlm.nih.gov/gene/15916
Idd20	Chromosome 6	http://www.ncbi.nlm.nih.gov/gene?term=Idd20
Idd21	Chromosome 18	http://www.ncbi.nlm.nih.gov/gene/492940
Idd21.1	Chromosome 18	http://www.ncbi.nlm.nih.gov/gene/100036455
Idd21.2	Chromosome 18	http://www.ncbi.nlm.nih.gov/gene/100036335
Idd21.3	Chromosome 18	http://www.ncbi.nlm.nih.gov/gene/100035246
Idd22	Chromosome 8	
Idd23	Chromosome 17	http://www.ncbi.nlm.nih.gov/gene?term=Idd23
Idd25	Chromosome 4	
Idd26	Chromosome 1	
Idd27	Chromosome 7	
Pik3cg	Chromosome 12	http://www.ncbi.nlm.nih.gov/gene/30955
