# Supplementary material for: PI3Kγ Deficient NOD-Mice Are Protected from Diabetes by Restoring the Balance of Regulatory to Effector-T-Cells
Source: PLoS One. 2017 Jan 12;12(1):e0169695. doi: 10.1371/journal.pone.0169695 (PMC5231340; doi:10.1371/journal.pone.0169695)
Supplement: S1 File — A. Description of the Marker-Assisted Accelerated Backcrossing technology used to generate the new NOD.PI3Kγ-/- mouse. B. Data from the Marker-Assisted-Accelerated-Backcrossing-program at Charles-River Laboratory that shows the scan of a panel of 384 single-nucleotide-polymorphisms (SNP) throughout of the genome of NOD.PI3Kγ-/- mouse. (PDF) [file pone.0169695.s003.pdf]

Marker-Assisted Accelerated Backcrossing (MAX-BAX®)
Service used from Charles River

MAX-BAX® Technology

Congenic strains are essential tools in biomedical research that allow reduction of genetic variability as they are identical at all genetic loci except for one; that differing locus is usually the transgene or knockout region of interest. The traditional, random backcrossing methods takes up to 2.5 year and 10 generations to produce a congenic strain. Selectively breeding individuals containing more of the recipient genome from each generation allows for accelerated congenic strain production. Those animals carrying the locus of interest with the highest percentage of recipient versus donor strain DNA are preferentially bred.
Genetic markers mapped to specific locations on each chromosome are used to evaluate strain-specific genomic polymorphism. The MAX-BAXZ technology uses mouse single nucleotide polymorphism (SNP) panel, composed of 384 carefully selected SNP markers. It was designed to maximize polymorphism between common inbred strains and provide even coverage of the mouse genome. Markers are spaced at approximately 7 Mbp intervals, and about half of the markers will be polymorphic between any inbred or outbred strains, allowing the same panel to be used for any donor and recipient strains.

The testing for the 384 SNP marker panel is performed on a microarray platform using robust fluorescence-based SNP genotyping assays. Results yield a defined analysis of the genome in question, and a preferred breeding rank is determined for all test individuals. The entire genome is analyzed at each generation, which may make unexpected genetic variation or breeding errors easier to detect.

Strain: PI3K x NOD (N6)
Client: Brigham Women's
Date Received: 27 April 2011

Mouse 384 SNP Panel

charles river

array		Marker	SNP ID	Chr	Start (bp)	591	585	624	623	587	NOD/LtJ	C57BL/6J
1	1	Chr1-1	rs13475705	1	5585594	1	1	1	1	1	1	1
1	2	Chr1-2	rs13475709	1	6646045	1	1	1	1	1	1	0
2	3	Chr1-3	rs13475732	1	13768325	1	1	1	1	1	1	0
2	4	Chr1-4	rs4222181	1	20804718	1	1	1	1	1	1	0
3	5	Chr1-5	rs6401470	1	27672455	1	1	1	1	1	1	0
3	6	Chr1-6	rs4222269	1	34601201	1	1	1	1	1	1	0
4	7	Chr1-7	rs13475847	1	45969220	1	0.5	1	1	1	1	0
4	8	Chr1-8	rs32602884	1	47261017	1	0.5	1	1	1	1	0
5	9	Chr1-9	rs6239834	1	63358048	1	0.5	0.5	0.5	1	1	0
5	10	Chr1-10	rs13475919	1	73020555	1	0.5	0.5	1	1	1	0
6	11	Chr1-11	rs13475928	1	75522038	1	1	1	1	1	1	1
6	12	Chr1-12	rs3698264	1	79343179	1	1	1	1	1	1	1
7	13	Chr1-13	rs4222516	1	92678218	1	1	1	1	1	1	1
7	14	Chr1-14	rs13475979	1	93218895	0.5	0.5	0.5	1	1	1	0
8	15	Chr1-15	rs13476024	1	105452781	0.5	0.5	0.5	1	1	1	0
8	16	Chr1-16	rs6168275	1	113643051	1	1	1	1	1	1	1
9	17	Chr1-17	rs13476064	1	116488503	0.5	0.5	0.5	1	1	1	0
65	18	Chr1-18	rs6242814	1	118897095	1	1	1	1	1	1	1
66	19	Chr1-19	rs4222623	1	133564494	1	1	1	1	1	1	1
10	20	Chr1-20	rs13476119	1	136107291	0.5	0.5	0.5	1	1	1	0
67	21	Chr1-21	rs13476155	1	146800337	1	1	1	1	1	1	0
11	22	Chr1-22	rs13476192	1	157761942	1	1	1	1	1	1	0
68	23	Chr1-23	rs13476195	1	158916123	1	1	1	1	1	1	0
12	24	Chr1-24	rs13476223	1	169640436	1	1	1	1	1	1	0
69	25	Chr1-25	rs3022871	1	174888139	1	1	1	1	1	1	0
13	26	Chr1-26	rs13476267	1	182213911	1	1	1	1	1	1	1
14	27	Chr1-27	rs13476286	1	187711002	1	1	1	1	1	1	0
70	28	Chr1-28	rs13476294	1	190268439	1	1	1	1	1	1	0
71	29	Chr2-1	rs13476337	2	7991482	1	1	1	1	1	1	0
15	30	Chr2-2	rs13476344	2	9314267	1	1	1	1	1	1	1
16	31	Chr2-3	rs13476350	2	11707492	1	1	1	1	1	1	1
72	32	Chr2-4	rs6407520	2	20299013	1	1	1	1	1	1	0
17	33	Chr2-5	rs13476383	2	23294859	1	1	1	1	1	1	1
129	34	Chr2-6	rs33640786	2	36540166	1	1	1	1	1	1	0
18	35	Chr2-7	rs13476465	2	43647240	1	1	1	1	1	1	1
19	36	Chr2-8	rs6265423	2	47020132	1	1	1	1	1	1	0
130	37	Chr2-9	rs13476491	2	50808753	1	1	1	1	1	1	0
20	38	Chr2-10	rs13476537	2	62483559	1	1	1	1	1	1	0
131	39	Chr2-11	rs6314726	2	63950900	1	1	1	1	1	1	0
21	40	Chr2-12	rs6353593	2	68262871	1	1	1	1	1	1	0
132	41	Chr2-13	rs13476583	2	75747539	1	1	1	1	1	1	1
22	42	Chr2-14	rs13476621	2	87193247	1	1	1	1	1	1	0
133	43	Chr2-15	rs6170908	2	94377940	1	1	1	1	1	1	0
23	44	Chr2-16	rs13476689	2	107305294	1	1	1	1	1	1	0
134	45	Chr2-17	rs4223383	2	109740681	1	1	1	1	1	1	0
135	46	Chr2-18	rs3022895	2	119407520	1	1	1	1	1	1	0
24	47	Chr2-19	rs13476740	2	121412769	1	1	1	1	1	1	1
136	48	Chr2-20	rs13465151	2	130507288	1	1	1	1	1	1	1
25	49	Chr2-21	rs13476787	2	134965497	1	1	1	1	1	1	0
9	50	Chr2-22	rs3022909	2	145107384	1	1	1	1	1	1	1
26	51	Chr2-23	rs13476846	2	152857172	1	1	1	1	1	1	0
10	52	Chr2-24	rs6204809	2	161566039	1	1	1	1	1	1	1
27	53	Chr2-25	rs13476894	2	165876927	1	1	1	1	1	1	0
11	54	Chr2-26	rs13476923	2	172651890	1	1	1	1	1	1	1
28	55	Chr3-1	rs13476950	3	3785419	1	1	1	1	1	1	1
12	56	Chr3-2	rs13476956	3	5370727	1	1	1	1	1	1	0
13	57	Chr3-3	rs6410894	3	19731453	1	1	1	1	1	1	0
29	58	Chr3-4	rs13477017	3	22507002	1	1	1	1	1	1	1
14	59	Chr3-5	rs6371982	3	34034360	1	1	1	1	1	1	1
30	60	Chr3-6	rs13477089	3	46190285	1	1	1	1	1	1	0
31	61	Chr3-7	rs13477113	3	53073655	1	1	1	1	1	1	0
15	62	Chr3-8	rs13477160	3	64739341	1	1	1	1	1	1	1
32	63	Chr3-9	rs13477200	3	75061692	1	1	1	1	1	1	0
16	64	Chr3-10	rs4223998	3	78920216	1	1	1	1	1	1	0
33	65	Chr3-11	rs13477251	3	88658602	1	1	1	1	1	1	1
73	66	Chr3-12	rs13477256	3	90488442	1	1	1	1	1	1	0
34	67	Chr3-13	rs13459184	3	94926333	1	1	1	1	1	1	0
74	68	Chr3-14	rs13459070	3	104607548	1	1	1	1	1	1	1
35	69	Chr3-15	rs13477351	3	114211027	1	1	1	1	1	1	0
75	70	Chr3-16	rs13477364	3	118290075	1	1	1	1	1	1	0
36	71	Chr3-17	rs4221957	3	129527944	1	1	1	1	1	1	1
76	72	Chr3-18	rs13477433	3	136253587	0.5	1	1	1	0.5	1	0
77	73	Chr3-19	rs30057041	3	146256384	0.5	1	1	0.5	1	1	0
37	74	Chr3-20	rs30213296	3	148269916	0.5	1	1	0.5	1	1	0
78	75	Chr3-21	rs13477512	3	156610793	0.5	1	1	0.5	1	1	0
38	76	Chr3-22	rs13477528	3	159475020	0.5	1	1	0.5	1	1	0
79	77	Chr4-1	rs13477541	4	6320883	1	1	1	1	1	1	0
39	78	Chr4-2	rs13477561	4	11507153	1	1	1	1	1	1	0
80	79	Chr4-3	rs32479301	4	21218277	1	1	1	1	1	1	0

Strain: PI3K x NOD (N6)
Client: Brigham Women's
Date Received: 27 April 2011

Mouse 384 SNP Panel

array		Marker	SNP ID	Chr	Start (bp)	591	585	624	623	587	NOD/LtJ	C57BL/6J
137	80	Chr4-4	rs13477633	4	33094104	1	1	1	1	1	1	1
40	81	Chr4-5	rs13459075	4	41664425	1	1	1	1	1	1	1
138	82	Chr4-6	rs3706700	4	49109938	1	1	1	1	1	1	1
41	83	Chr4-7	rs6204339	4	51949195	1	1	1	1	1	1	0
42	84	Chr4-8	rs13477724	4	57442782	1	1	1	1	1	1	1
139	85	Chr4-9	rs6374954	4	63036830	1	1	1	0.5	1	1	0
43	86	Chr4-10	rs13477743	4	64461467	nr	nr	nr	nr	nr	1	0
140	87	Chr4-11	rs16265	4	77612028	1	1	1	1	1	1	1
44	88	Chr4-12	rs13477799	4	79853958	1	1	1	1	1	1	1
141	89	Chr4-13	rs6206306	4	90175455	1	1	1	1	1	1	0
45	90	Chr4-14	rs13477859	4	96869172	1	1	1	1	1	1	0
142	91	Chr4-15	rs6324470	4	105650718	1	1	1	1	1	1	0
46	92	Chr4-16	rs6381371	4	115708649	1	1	1	1	1	1	0
143	93	Chr4-17	rs13477947	4	120691835	1	1	1	1	1	1	0
47	94	Chr4-18	rs13477970	4	127258946	1	1	1	1	1	1	1
48	95	Chr4-19	rs13459077	4	132419164	1	1	1	0.5	1	1	0
144	96	Chr4-20	rs4224824	4	135004661	1	1	1	0.5	1	1	1
49	97	Chr4-21	rs13478014	4	138566497	1	1	1	1	1	1	1
17	98	Chr4-22	rs4224947	4	149023755	1	1	1	1	1	1	0
50	99	Chr4-23	rs13478063	4	152699720	1	1	1	1	1	1	0
18	100	Chr4-24	rs13478076	4	155236792	1	1	1	1	1	1	1
19	101	Chr5-1	rs6245801	5	7461340	1	1	1	1	1	1	0
51	102	Chr5-2	rs13481342	5	12837850	1	1	1	1	1	1	0
20	103	Chr5-3	rs13478131	5	20532162	1	1	1	1	1	1	0
52	104	Chr5-4	rs3023621	5	28888081	1	1	1	1	1	1	0
21	105	Chr5-5	rs13478174	5	33329619	1	1	1	1	1	1	1
53	106	Chr5-6	rs6276465	5	42600456	nr	nr	nr	nr	nr	1	1
22	107	Chr5-7	rs3023045	5	50966233	1	1	1	1	1	1	0
54	108	Chr5-8	rs13478269	5	58107090	1	1	1	1	1	1	0
23	109	Chr5-9	rs13478311	5	68029080	1	1	1	1	1	1	0
55	110	Chr5-10	rs6235071	5	73033762	1	1	1	1	1	1	0
24	111	Chr5-11	rs13478339	5	78253018	1	1	1	1	1	1	0
56	112	Chr5-12	rs3688741	5	86787409	1	1	1	1	1	1	0
81	113	Chr5-13	rs13478391	5	91904489	1	1	1	1	1	1	0
57	114	Chr5-14	rs13478419	5	100829662	1	1	1	1	1	1	1
82	115	Chr5-15	rs13478434	5	104572764	1	1	1	1	1	1	1
58	116	Chr5-16	rs13478473	5	114660997	1	1	1	1	1	1	1
83	117	Chr5-17	rs13478487	5	119758227	1	1	1	1	1	1	1
59	118	Chr5-18	rs13478499	5	123738720	0.5	1	1	1	0.5	1	0
84	119	Chr5-19	rs13478533	5	131210820	1	1	1	1	1	1	0
60	120	Chr5-20	rs13478547	5	137222873	1	1	1	1	1	1	0
85	121	Chr5-21	rs3023062	5	145631811	1	1	1	1	1	1	1
61	122	Chr5-22	rs13478595	5	151148537	1	1	1	1	1	1	0
86	123	Chr6-1	rs6206775	6	7458991	1	1	1	1	1	1	1
62	124	Chr6-2	rs13478641	6	16011005	1	1	1	1	1	1	0
87	125	Chr6-3	rs13478656	6	21893927	1	1	1	1	1	1	0
63	126	Chr6-4	rs6218880	6	25230499	1	1	1	1	1	1	0
88	127	Chr6-5	rs13478698	6	32910258	1	1	1	1	1	1	0
64	128	Chr6-6	rs6238771	6	45196664	1	1	1	1	1	1	0
145	129	Chr6-7	rs13478754	6	51720316	1	1	1	1	1	1	1
65	130	Chr6-8	rs13478768	6	55504762	1	1	1	1	1	1	0
146	131	Chr6-9	rs13478785	6	60941037	1	1	1	1	1	1	1
66	132	Chr6-10	rs6246241	6	71720626	1	1	1	1	1	1	0
147	133	Chr6-11	rs13478834	6	76774055	1	1	1	1	1	1	1
67	134	Chr6-12	rs30216797	6	84079021	1	1	1	1	1	1	1
148	135	Chr6-13	rs13478887	6	91019207	1	1	1	1	1	1	0
68	136	Chr6-14	rs13478917	6	97419188	1	1	1	1	1	1	0
149	137	Chr6-15	rs6292642	6	104503229	1	1	1	1	1	1	1
69	138	Chr6-16	rs13478979	6	113251663	1	1	1	1	1	1	0
150	139	Chr6-17	rs13479001	6	119516194	1	1	1	1	1	1	1
70	140	Chr6-18	rs13479044	6	131637351	1	1	1	1	1	1	1
151	141	Chr6-19	rs4226318	6	133057628	1	0.5	0.5	0.5	1	1	0
71	142	Chr6-20	rs13479078	6	139834735	1	1	1	1	1	1	0
152	143	Chr6-21	rs3090690	6	146408574	1	1	1	1	1	1	0
72	144	Chr6-22	rs6328711	6	148826464	1	1	1	1	1	1	1
25	145	Chr7-1	rs13479114	7	7092609	1	1	1	1	1	1	0
73	146	Chr7-2	rs13479137	7	16488107	1	1	1	1	1	1	1
26	147	Chr7-3	rs13479145	7	19988355	1	1	1	1	1	1	0
74	148	Chr7-4	rs13479175	7	31087252	1	1	1	1	1	1	1
27	149	Chr7-5	rs6239372	7	35483870	1	1	1	1	1	1	0
75	150	Chr7-6	rs6206014	7	47861154	1	1	1	1	1	1	0
28	151	Chr7-7	rs13479233	7	55071694	1	1	1	1	1	1	0
76	152	Chr7-8	rs13479271	7	64748561	1	1	1	1	1	1	1
29	153	Chr7-9	rs13479292	7	69695364	1	1	1	1	1	1	1
77	154	Chr7-10	rs13479342	7	82114282	1	1	1	1	1	1	0
30	155	Chr7-11	rs6373032	7	83914252	1	1	1	1	1	1	1
78	156	Chr7-12	rs6344724	7	91156584	1	1	1	1	1	1	0
31	157	Chr7-13	rs13479402	7	99329410	1	1	1	1	1	1	1
79	158	Chr7-14	rs13479413	7	102506944	1	1	1	1	1	1	0

Strain: PI3K x NOD (N6)
Client: Brigham Women's
Date Received: 27 April 2011

Mouse 384 SNP Panel

charles river

array		Marker	SNP ID	Chr	Start (bp)	591	585	624	623	587	NOD/LtJ	C57BL/6J
32	159	Chr7-15	rs6357312	7	109389815	1	1	1	1	1	1	1
80	160	Chr7-16	rs13479469	7	121418263	1	1	1	1	1	1	0
89	161	Chr7-17	rs13459101	7	125673109	1	1	1	1	1	1	1
81	162	Chr7-18	rs6252075	7	134800915	1	1	0.5	0.5	1	1	0
90	163	Chr7-19	rs13479537	7	140062817	1	1	1	1	1	1	1
82	164	Chr7-20	rs13479556	7	145553382	1	1	1	1	1	1	0
91	165	Chr7-21	rs4226997	7	152082670	1	1	1	1	1	1	0
83	166	Chr7-22	rs13479572	7	152494066	1	1	1	1	1	1	0
92	167	Chr8-1	rs13479602	8	9252746	1	1	1	1	1	1	1
84	168	Chr8-2	rs30244710	8	15526052	1	1	1	1	1	1	0
93	169	Chr8-3	rs13479633	8	19452131	1	1	1	1	1	1	0
85	170	Chr8-4	rs13479641	8	24972916	1	1	1	1	1	1	1
86	171	Chr8-5	rs13479681	8	32848679	1	1	1	1	1	1	0
94	172	Chr8-6	rs32612161	8	34056457	1	1	1	1	1	1	0
87	173	Chr8-7	rs13479694	8	35274784	1	1	1	1	1	1	0
95	174	Chr8-8	rs13479752	8	51299032	1	1	1	1	1	1	1
88	175	Chr8-9	rs13479785	8	61639733	1	1	1	1	1	1	0
89	176	Chr8-10	rs13479807	8	68141750	1	1	1	1	1	1	0
96	177	Chr8-11	rs13479815	8	71137663	1	1	1	1	1	1	0
153	178	Chr8-12	rs4227253	8	77650956	1	1	1	1	1	1	1
90	179	Chr8-13	rs13479865	8	86257018	1	1	1	1	1	1	0
154	180	Chr8-14	rs13479892	8	90842707	1	1	1	1	1	1	0
91	181	Chr8-15	rs13479948	8	102254876	1	1	1	1	1	1	0
155	182	Chr8-16	rs13479961	8	105921528	1	1	1	1	1	1	1
92	183	Chr8-17	rs6409975	8	118561265	1	1	1	1	1	1	1
156	184	Chr8-18	rs13480010	8	119637332	1	1	1	1	1	1	0
93	185	Chr8-19	rs13480031	8	127756867	1	1	1	1	1	1	0
157	186	Chr8-20	rs13480045	8	130889097	1	1	1	1	1	1	1
158	187	Chr9-1	rs6167568	9	6643072	1	1	1	1	1	1	1
94	188	Chr9-2	rs4137000	9	13259371	1	1	1	1	1	1	0
159	189	Chr9-3	rs6181976	9	23780477	1	1	1	1	1	1	1
95	190	Chr9-4	rs6222175	9	31523010	1	1	1	1	1	1	1
160	191	Chr9-5	rs30435524	9	34769349	1	1	1	1	1	1	1
96	192	Chr9-6	rs13480166	9	43879831	1	1	1	1	1	1	0
33	193	Chr9-7	rs3023210	9	48612132	1	1	1	1	1	1	0
97	194	Chr9-8	rs13480205	9	54377877	1	1	1	1	1	1	0
34	195	Chr9-9	rs13480238	9	63420684	1	1	1	1	1	1	0
98	196	Chr9-10	rs13480264	9	70338300	1	1	1	1	1	1	0
35	197	Chr9-11	rs6299479	9	75526199	1	1	1	1	1	1	1
99	198	Chr9-12	rs13480317	9	85678310	1	1	1	1	1	1	1
36	199	Chr9-13	rs33103151	9	90578038	1	1	1	1	1	1	1
100	200	Chr9-14	rs13480382	9	102330061	1	1	1	0.5	1	1	0
37	201	Chr9-15	rs13480400	9	106550262	1	1	1	1	1	1	0
101	202	Chr9-16	rs13480428	9	113200235	1	1	1	1	1	1	0
38	203	Chr9-17	rs6372658	9	117873471	1	1	1	1	1	1	1
102	204	Chr9-18	rs6299531	9	122834341	1	1	1	1	1	1	1
39	205	Chr10-1	rs13480488	10	8964116	1	1	1	1	1	1	1
103	206	Chr10-2	rs13480510	10	15801686	1	1	1	1	1	1	1
40	207	Chr10-3	rs13459119	10	20045489	1	1	1	1	1	1	0
104	208	Chr10-4	rs13480570	10	30725948	1	1	1	1	1	1	0
97	209	Chr10-5	rs13480581	10	38685357	1	1	1	1	1	1	1
105	210	Chr10-6	rs29327991	10	38770868	1	1	1	1	nr	1	1
98	211	Chr10-7	rs6382436	10	48405529	1	1	1	1	1	1	1
106	212	Chr10-8	rs29364962	10	57191368	1	1	1	1	1	1	1
99	213	Chr10-9	rs13480628	10	66700922	1	1	1	1	1	1	0
107	214	Chr10-10	rs13480641	10	69476909	1	1	1	1	1	1	1
100	215	Chr10-11	rs13480657	10	76175591	1	1	1	1	1	1	0
108	216	Chr10-12	rs13480660	10	77019281	1	1	1	1	1	1	0
109	217	Chr10-13	rs13480693	10	88365917	1	1	1	1	1	1	0
101	218	Chr10-14	rs13480706	10	91625255	1	1	1	1	1	1	0
110	219	Chr10-15	rs13480740	10	103515832	1	1	1	1	1	1	1
102	220	Chr10-16	rs13480749	10	105437651	1	1	1	1	1	1	1
111	221	Chr10-17	rs13480762	10	110233294	1	1	1	1	1	1	1
103	222	Chr10-18	rs29329210	10	112836909	1	1	1	1	1	1	1
112	223	Chr10-19	rs13480793	10	120465110	1	1	1	1	1	1	0
104	224	Chr10-20	rs3676330	10	127647104	1	1	1	1	1	1	1
161	225	Chr11-1	rs13480864	11	9452733	1	1	1	0.5	1	1	1
113	226	Chr11-2	rs6211409	11	14121211	1	1	1	1	1	1	0
162	227	Chr11-3	rs13480906	11	21938817	1	1	1	1	1	1	1
114	228	Chr11-4	rs13480937	11	30059387	1	1	1	1	1	1	0
163	229	Chr11-5	rs13480971	11	37065962	1	1	1	1	1	1	0
115	230	Chr11-6	rs13480992	11	45632883	1	1	1	1	1	1	1
164	231	Chr11-7	rs13459130	11	54080664	1	1	1	1	1	1	1
116	232	Chr11-8	rs13481048	11	58775634	1	1	1	1	1	1	1
165	233	Chr11-9	rs13481070	11	64909099	1	1	1	1	1	1	0
117	234	Chr11-10	rs6197743	11	69990882	1	1	1	1	1	1	1
166	235	Chr11-11	rs13481105	11	75460557	1	1	1	1	1	1	0
118	236	Chr11-12	rs13481123	11	82070986	1	1	1	1	1	1	1
167	237	Chr11-13	rs13481154	11	90203180	1	1	1	1	1	1	0

Strain: PI3K x NOD (N6)
Client: Brigham Women's
Date Received: 27 April 2011

Mouse 384 SNP Panel

charles river

array		Marker	SNP ID	Chr	Start (bp)	591	585	624	623	587	NOD/LtJ	C57BL/6J
119	238	Chr11-14	rs13481176	11	97413258	1	1	1	1	1	1	0
168	239	Chr11-15	rs13481201	11	103038835	1	1	1	1	1	1	1
120	240	Chr11-16	rs13481219	11	107795749	1	1	1	0.5	1	1	0
41	241	Chr11-17	rs13481249	11	116402897	1	1	1	1	1	1	0
121	242	Chr11-18	rs6280170	11	120340622	1	1	1	1	1	1	1
122	243	Chr12-1	rs29199985	12	5407521	1	1	1	1	1	1	1
42	244	Chr12-2	rs13481294	12	9577137	1	1	1	1	1	1	0
123	245	Chr12-3	rs13481301	12	11672960	1	1	0.5	0.5	1	1	0
43	246	Chr12-4	rs13481350	12	25341610	1	1	0.5	0.5	1	1	1
124	247	Chr12-5	rs13481372	12	31085307	1	1	1	1	1	1	1
44	248	Chr12-6	rs29204715	12	34740768	0.5	0.5	0.5	0.5	0.5	1	1
125	249	Chr12-7	rs13481406	12	40661366	0.5	0.5	0.5	0.5	0.5	1	0
45	250	Chr12-8	rs13481431	12	47902411	1	1	1	1	1	1	0
126	251	Chr12-9	rs33845879	12	55866589	0.5	0.5	0.5	0.5	0.5	1	0
46	252	Chr12-10	rs13481489	12	62634264	0.5	0.5	0.5	0.5	0.5	1	1
127	253	Chr12-11	rs13481515	12	71027098	1	1	1	1	1	1	1
47	254	Chr12-12	rs8259450	12	75043079	0.5	0.5	0.5	1	0.5	1	1
128	255	Chr12-13	rs29152777	12	83974583	1	1	1	1	1	1	0
48	256	Chr12-14	rs13481583	12	91589325	1	1	1	1	1	1	0
129	257	Chr12-15	rs13481618	12	103766712	1	1	1	1	1	1	1
105	258	Chr12-16	rs13481627	12	106109309	1	1	0.5	1	1	1	0
130	259	Chr12-17	rs6176416	12	112919383	1	1	1	1	1	1	0
106	260	Chr12-18	rs13481659	12	114730692	1	1	1	1	1	1	1
107	261	Chr13-1	rs6277459	13	9749397	1	1	1	1	1	1	0
131	262	Chr13-2	rs13481708	13	16818885	1	1	1	1	1	1	0
108	263	Chr13-3	rs13481718	13	19919702	1	1	1	1	1	1	1
132	264	Chr13-4	rs13481740	13	30769382	1	1	1	1	1	1	1
109	265	Chr13-5	rs13481767	13	37610928	1	1	1	1	1	1	1
133	266	Chr13-6	rs3688207	13	45454657	1	1	1	1	1	1	1
110	267	Chr13-7	rs29533855	13	47339591	1	1	1	1	1	1	0
134	268	Chr13-8	rs13481828	13	58152623	1	1	1	1	1	1	1
111	269	Chr13-9	rs29879705	13	61062538	1	1	1	1	1	1	0
135	270	Chr13-10	rs13481866	13	70083586	1	1	1	1	1	1	0
112	271	Chr13-11	rs13481887	13	76470779	1	1	1	1	1	1	0
136	272	Chr13-12	rs6245977	13	85699164	1	1	1	1	1	1	1
169	273	Chr13-13	rs13481942	13	92011615	1	1	1	1	1	1	1
137	274	Chr13-14	rs13481967	13	99462341	1	1	1	1	1	1	0
170	275	Chr13-15	rs13481979	13	103343044	1	1	1	1	1	1	1
138	276	Chr13-16	rs30072381	13	109231972	1	1	1	0.5	1	1	1
171	277	Chr13-17	rs13482027	13	116327508	1	1	1	1	1	1	0
139	278	Chr13-18	rs6412462	13	119764543	1	1	1	1	1	1	0
172	279	Chr14-1	rs13482048	14	9848946	1	1	1	1	1	1	0
140	280	Chr14-2	rs13482072	14	16563741	1	1	1	1	1	1	1
173	281	Chr14-3	rs13482097	14	24572106	1	1	1	1	1	1	0
141	282	Chr14-4	rs13482110	14	28588757	1	1	1	0.5	1	1	0
174	283	Chr14-5	rs13482135	14	36200743	1	1	1	1	1	1	1
142	284	Chr14-6	rs13482143	14	40378548	1	1	1	1	1	1	0
175	285	Chr14-7	rs13482176	14	51060859	1	1	1	1	1	1	1
143	286	Chr14-8	rs13482202	14	59404366	1	1	1	1	1	1	1
176	287	Chr14-9	rs3725809	14	64867471	1	1	1	1	1	1	1
144	288	Chr14-10	rs13482246	14	71920113	1	1	1	1	1	1	0
49	289	Chr14-11	rs13482266	14	79556856	1	1	1	1	1	1	1
145	290	Chr14-12	rs13482284	14	86615894	1	1	1	1	1	1	0
50	291	Chr14-13	rs6334493	14	100286465	1	1	1	1	1	1	1
146	292	Chr14-14	rs13482339	14	101401164	1	1	1	1	1	1	1
51	293	Chr14-15	rs13482368	14	110905420	1	1	1	1	1	1	0
147	294	Chr14-16	rs13482393	14	119128126	1	1	1	1	1	1	0
52	295	Chr14-17	rs4230603	14	121343810	1	1	1	1	1	1	0
148	296	Chr14-18	rs4230609	14	121682448	1	1	1	1	1	1	1
53	297	Chr15-1	rs13482429	15	10505267	1	1	1	0.5	1	1	0
149	298	Chr15-2	rs13482442	15	14008099	1	1	1	1	1	1	0
54	299	Chr15-3	rs13482477	15	22448960	1	1	1	1	1	1	1
150	300	Chr15-4	rs13482497	15	28251498	1	1	1	1	1	1	1
55	301	Chr15-5	rs6153770	15	35056255	1	1	1	1	1	1	0
151	302	Chr15-6	rs13482536	15	41700828	1	1	1	1	1	1	0
56	303	Chr15-7	rs13482563	15	48437257	1	1	1	1	1	1	1
152	304	Chr15-8	rs13482572	15	50042767	1	1	1	1	1	1	1
153	305	Chr15-9	rs13482590	15	54944934	1	1	1	1	1	1	1
113	306	Chr15-10	rs13482618	15	65690150	1	1	1	1	1	1	0
154	307	Chr15-11	rs6169611	15	70046283	1	1	1	1	1	1	0
114	308	Chr15-12	rs4230847	15	78605289	1	1	1	1	1	1	1
155	309	Chr15-13	rs13482682	15	84449486	1	1	1	1	1	1	0
115	310	Chr15-14	rs13482701	15	89705712	1	1	1	0.5	1	1	0
156	311	Chr15-15	rs13482713	15	92378560	1	1	1	1	1	1	0
116	312	Chr15-16	rs13482744	15	101380978	1	1	1	1	1	1	1
157	313	Chr16-1	rs4152386	16	3987018	1	1	1	1	1	1	1
117	314	Chr16-2	rs4154322	16	6124729	1	1	1	1	1	1	1
158	315	Chr16-3	rs4163487	16	13677988	1	1	1	1	1	1	1
118	316	Chr16-4	rs4165081	16	19883172	1	1	1	1	1	1	1

Strain: PI3K x NOD (N6)
Client: Brigham Women's
Date Received: 27 April 2011

Mouse 384 SNP Panel

charles river

array		Marker	SNP ID	Chr	Start (bp)	591	585	624	623	587	NOD/LtJ	C57BL/6J
159	317	Chr16-5	rs4165334	16	23467678	1	1	1	1	1	1	0
119	318	Chr16-6	rs3666382	16	34212148	1	1	1	1	1	1	1
160	319	Chr16-7	rs4178870	16	42031091	1	1	1	1	1	1	1
120	320	Chr16-8	rs4183630	16	47373559	1	1	1	1	1	1	1
161	321	Chr16-9	rs4186801	16	51465652	1	1	1	1	1	1	0
177	322	Chr16-10	rs4194384	16	62450621	1	1	1	1	1	1	1
162	323	Chr16-11	rs4198331	16	68115290	1	1	1	1	1	1	1
178	324	Chr16-12	rs4208724	16	78685266	1	1	1	1	1	1	0
163	325	Chr16-13	rs4212481	16	85641478	1	1	1	1	1	1	0
179	326	Chr16-14	rs4220668	16	94891033	1	1	1	1	1	1	0
180	327	Chr17-1	rs13482863	17	9351887	1	1	1	1	1	1	1
164	328	Chr17-2	rs13482864	17	9565936	1	1	1	1	1	1	1
181	329	Chr17-3	rs13482921	17	24109788	1	1	1	1	1	1	1
165	330	Chr17-4	rs6358703	17	28916155	1	1	1	1	1	1	0
182	331	Chr17-5	rs3023110	17	33517718	1	1	1	1	1	1	1
166	332	Chr17-6	rs13482980	17	40555049	1	1	1	1	1	1	1
183	333	Chr17-7	rs4231532	17	47957927	1	1	1	1	1	1	1
167	334	Chr17-8	rs13483029	17	52485097	1	1	1	1	1	1	0
184	335	Chr17-9	rs13483058	17	61680643	1	1	1	1	1	1	0
168	336	Chr17-10	rs6399089	17	68482570	1	1	1	1	1	1	1
57	337	Chr17-11	rs13483117	17	79240322	1	1	1	1	1	1	0
169	338	Chr17-12	rs6158628	17	82052484	1	1	1	1	1	1	1
58	339	Chr17-13	rs13483160	17	90557266	1	1	1	1	1	1	0
170	340	Chr17-14	rs13483174	17	93935624	1	1	1	1	1	1	0
59	341	Chr18-1	rs6224965	18	5079849	1	1	1	1	1	1	1
171	342	Chr18-2	rs13483238	18	19542414	1	1	1	1	1	1	0
60	343	Chr18-3	rs13483253	18	23959739	1	1	1	1	1	1	0
172	344	Chr18-4	rs13483278	18	30610888	1	1	1	1	1	1	0
61	345	Chr18-5	rs13483308	18	38428528	1	1	1	1	1	1	1
173	346	Chr18-6	rs6157163	18	44702560	1	1	1	1	1	1	0
174	347	Chr18-7	rs13483358	18	50847061	1	1	1	1	1	1	0
62	348	Chr18-8	rs13483365	18	53553182	1	1	1	1	1	1	1
175	349	Chr18-9	rs6338896	18	58717533	1	1	1	1	1	1	1
63	350	Chr18-10	rs13483401	18	63939575	1	1	1	1	1	1	1
176	351	Chr18-11	rs29768513	18	70944925	1	0.5	0.5	0.5	1	1	0
64	352	Chr18-12	rs3705890	18	77108823	1	1	1	1	1	1	1
177	353	Chr18-13	rs13483466	18	82409998	1	1	1	1	1	1	1
121	354	Chr18-14	rs13483497	18	90273335	1	1	1	1	1	1	1
178	355	Chr19-1	rs13483526	19	10670708	1	1	1	1	1	1	0
122	356	Chr19-2	rs13483548	19	17844682	1	1	1	1	1	1	0
179	357	Chr19-3	rs4232111	19	24047254	1	1	1	1	1	1	1
123	358	Chr19-4	rs6238842	19	30355837	1	1	1	1	1	1	1
180	359	Chr19-5	rs13483605	19	35692493	1	1	1	1	1	1	1
124	360	Chr19-6	rs13483642	19	45215423	1	1	1	1	1	1	1
181	361	Chr19-7	rs13483665	19	51255209	1	1	1	1	1	1	0
125	362	Chr19-8	rs13483695	19	59315566	1	1	1	1	1	1	1
126	363	ChrX-1	rs13483704	X	7560445	1	1	1	1	1	1	1
182	364	ChrX-2	rs13483715	X	9788448	1	1	1	1	1	1	1
127	365	ChrX-3	rs13483718	X	10778885	1	1	1	1	1	1	1
183	366	ChrX-4	rs13483722	X	19873182	1	1	1	1	1	1	0
128	367	ChrX-5	rs13483730	X	39577867	1	1	1	1	1	1	1
184	368	ChrX-6	rs33872015	X	40335088	1	1	1	1	1	1	1
185	369	ChrX-7	rs13483756	X	50304196	1	1	1	1	1	1	1
185	370	ChrX-8	rs13483770	X	56488675	1	1	1	1	1	1	0
186	371	ChrX-9	rs13483807	X	66136453	1	1	1	1	1	1	0
186	372	ChrX-10	rs13483818	X	69187248	1	1	1	1	1	1	0
187	373	ChrX-11	rs13483863	X	80517265	1	1	1	1	1	1	1
187	374	ChrX-12	rs13483878	X	84490778	1	1	1	1	1	1	1
188	375	ChrX-13	rs13483898	X	95119353	1	1	1	1	1	1	1
188	376	ChrX-14	rs13483926	X	103283749	1	1	1	1	1	1	1
189	377	ChrX-15	rs13483954	X	112067339	nr	nr	nr	nr	nr	1	1
189	378	ChrX-16	rs13483990	X	126823460	1	1	1	1	1	1	0
190	379	ChrX-17	rs29264998	X	133125033	1	1	1	1	1	1	0
190	380	ChrX-18	rs13459159	X	136345220	1	1	1	1	1	1	0
191	381	ChrX-19	rs13484072	X	146748154	1	1	1	1	1	1	0
191	382	ChrX-20	rs13484075	X	149936656	1	1	1	1	1	1	0
192	383	ChrX-21	rs13484098	X	158023555	1	1	1	1	1	1	1
192	384	ChrX-22	rs13484113	X	165892683	1	1	1	1	1	1	0

% NOD:

591	585	624	623	587	NOD/LtJ	C57BL/6J
98.03	98.03	97.77	96.85	99.08	100.00	45.05

Average Percentage:

97.95
